# Beyond general food craving: sex differences in food-specific craving identified using item response theory

**DOI:** 10.3389/fpsyg.2025.1588999

**Published:** 2025-06-17

**Authors:** Daiil Jun, Sean Joo, Tera L. Fazzino

**Affiliations:** ^1^Department of Psychology, University of Kansas, Lawrence, KS, United States; ^2^Cofrin Logan Center for Addiction Research and Treatment, University of Kansas, Lawrence, KS, United States; ^3^Department of Educational Psychology, University of Kansas, Lawrence, KS, United States

**Keywords:** food craving, sex difference, item response theory, differential item functioning, chocolate, Food Craving Inventory

## Abstract

**Background:**

Food craving plays a significant role in food choice and excess energy intake. While prior research has predominantly examined food craving as a general construct (e.g., craving for foods overall), this approach may obscure important variability in craving for specific foods. The current study aimed to address this limitation by examining craving for specific foods (food-item level) and examining differences across sex in food item craving, while controlling for overall food craving.

**Methods:**

The sample (*N* = 583) was collected via crowdsourcing. The Food Craving Inventory was used to assess craving for 28 food items. Using item response theory (IRT), a partial credit model was employed to investigate which food item was easier or more commonly craved, while holding overall food craving level constant across participants. Differential item functioning (DIF) analysis identified sex differences in craving for specific food items, with effect sizes calculated to interpret the magnitude of DIF.

**Results:**

The partial credit model revealed that pizza and chocolate were the easiest or most commonly craved, while gravy and cornbread were the most difficult to crave. DIF analysis suggested that savory items were more difficult (or less commonly) craved among females with medium effect sizes (i.e., Cohen's D) ranging from 0.53 to 0.80, whereas sweets were more difficult for males to crave, with effect sizes ranging from 0.42 to 0.49.

**Conclusion:**

Findings indicated that food craving may vary depending on specific foods and sex.

## 1 Introduction

Food craving, defined as an strong desire to consume a food (White et al., 2002), has been suggested to play a key role in disordered eating behaviors (e.g., binge eating) as well as excess energy intake and related conditions (e.g., obesity) (Boswell and Kober, 2016; Stopyra et al., 2021; Ince et al., 2021; Yu et al., 2022). In the food craving literature, food craving has mostly been measured as a general construct that represents either trait or state-based craving for foods in general (Taylor, 2019; Meule, 2020). Trait-based food craving represents how individuals tend to respond to or experience food craving and is hypothesized to be an individual-level difference, with some individuals experiencing higher craving overall compared to others (Cepeda-Benito et al., 2000; Meule, 2020). In contrast, state-based food craving represents momentary or situation-based food craving, which is hypothesized to vary over time and is assumed to be influenced by environment/situational contexts (Cepeda-Benito et al., 2000; Richard et al., 2017; Pannicke et al., 2022). Trait and state-based craving constructs have contributed to identifying the role of elevated food craving as an individual and state-level risk factor in disordered eating behaviors (Leslie et al., 2018; Reents and Pedersen, 2021; Schaefer et al., 2023), which has been important in identifying intervention targets for craving-induced overeating and binge eating (Sun and Kober, 2020).

Despite the usefulness of prior work in identifying high general food craving as an individual-level vulnerability, research characterizing food craving as a general construct has employed an assumption that measured craving may be similarly applicable to all types of foods. However, research focusing on aggregate food craving may overlook important variability in craving for specific foods that may contribute to disordered eating and excess energy intake. Previous studies have suggested that foods with specific nutrient combinations (e.g., high fat and sodium; fat and sugar; carbohydrates and sodium), termed hyper-palatable foods (HPF), may have particularly strong reinforcing properties (DiFeliceantonio et al., 2018; Epstein et al., 2012; Fazzino et al., 2019), and thus may more easily trigger food wanting and craving relative to whole, fresh foods that come from nature (Fazzino, 2022). Although there have been limited studies examining how craving differs across specific food items, some empirical evidence has suggested that HPF may be more easily craved than non-HPF. For example, one prior study measured craving for specific food items and found that foods that would be considered HPF (e.g., hamburgers; pizza) were more strongly craved than non-HPF (e.g., fruits and vegetables) among adults with obesity and binge eating disorder (Reents and Pedersen, 2021). Also, a study using ecological momentary assessment found that craving for HPF (e.g., hamburgers) was more frequently reported than craving for fresh foods (e.g., vegetables) among adults with elevated BMIs relative to adults with lower BMIs (Roefs et al., 2019). These findings highlight the importance of measuring cravings for specific food items, as some types of food may be more strongly craved than others and may differentially influence food seeking and intake behavior.

Measuring general food craving may also obscure the heterogeneity of food cravings across different contexts and for different groups of people. For example, some prior literature has suggested that cravings among males may differ significantly from females, with hypothesized reasons spanning from physiological differences to potential differences in food effects due to sociocultural and/or psychological reasons (Hallam et al., 2016). In general in the craving literature, the prevailing view has been that that females may exhibit higher cravings for sweets (e.g., chocolate) whereas males may exhibit greater craving for savory foods (Asarian and Geary, 2013; Hallam et al., 2016; Meule, 2020). Specifically, previous studies typically compared males' and females' average scores for a single item or closely related food items, which prevented them from disentangling whether observed sex differences were due to craving for the particular food item assessed (e.g., chocolate) or reflective of higher general craving in one sex compared to the other (Anton et al., 2012; Chao et al., 2016; Imperatori et al., 2013; Meule and Hormes, 2015). However, few studies have directly investigated sex differences in cravings for specific foods in the past decade, and thus, the methodological limitations of these studies have remained largely unaddressed.

To address the limitations of the prior literature, the present study sought to examine differences in cravings for different types of foods among a general sample of adults. The study used the Food Craving Inventory (FCI), which assesses food craving across different types of foods (White et al., 2002) and facilitated the examination of food craving at the item-level using item response theory (IRT) analysis. The study also sought to examine differences in food craving across food items by sex using differential item functioning (DIF) analysis.

## 2 Materials and methods

### 2.1 Study procedures and participants

The study was reviewed and approved by the host university's Institutional Review Board. The study used Amazon Mechanical Turk (MTurk), an online crowdsourcing platform, and collected data in the spring of 2020. Participant recruitment was conducted in six batches to accommodate individuals with varying schedule availabilities. To be eligible, participants had to be aged 18–65, reside in the United States, possess an MTurk approval rating of ≥99%, and have completed ≥1,000 studies on MTurk. These criteria were selected to adhere to MTurk's data quality standards and to align with the study's focus on US food measures. All participants provided informed consent (*N* = 602). Ten participants provided poor-quality data (i.e., failed at least one of three attention check questions), and seven participants had missing values in the FCI, and were thus removed from the data prior to analysis.

Participants were asked to select from the following options: male; female; transgender, male to female; transgender, female to male; intersex; and prefer not to answer. Unfortunately, sex and gender identity were both included in the response options. To recognize this limitation and to best reflect participant responses, we included participants who selected the male (*n* = 308) or female (*n* = 275) sex options in the current analyses. We did not have sufficient statistical power to analyze the other groupings (*n* = 2 selected transgender) and therefore did not include these participants in the analyses. Therefore, the final sample that we analyzed was *N* = 583.

### 2.2 Measures

The Food Craving Inventory (FCI) was employed to assess participants' craving for specific foods (White et al., 2002). The FCI is a widely used self-report measure designed to capture the intensity and frequency of cravings for specific foods. The FCI consists of 28 items, with each item corresponding to a particular food (e.g., chocolate, fried chicken). Participants assessed the frequency of their cravings for each item over the past month using a 5-point Likert scale, ranging from 1 (never) to 5 (always/almost every day). Generally in the literature, the FCI has been used to measure overall craving for food items (aggregated), or craving for specific categories of foods (e.g., fast foods) (White et al., 2002; Anton et al., 2012; Chao et al., 2016). In this study, the FCI was administered once to all study participants. We employed IRT to examine how craving differed across the 28 individual food items in the FCI. The FCI has demonstrated good internal consistency (α = 0.93), acceptable test-retest reliability (α = 0.86), concurrent validity, and discriminant validity (White et al., 2002), suggesting it is a robust measure for assessing food cravings. In this study, the FCI showed a good internal consistency (α = 0.93).

### 2.3 Data analysis

IRT analysis was employed to examine the characteristics of items and psychometric properties. Furthermore, DIF analysis was carried out to examine potential item bias against sex for the FCI. Analyses were conducted using the “mirt” package in R (Chalmers, 2012). IRT uses response to all FCI items to estimate individuals' latent food craving level while simultaneously determining the unique characteristics of each item (e.g., item difficulty).

Specifically, the partial credit model (PCM) was used to estimate item difficulty parameters. The PCM was specifically developed to analyze polytomous response data including Likert scale (Masters, 2016). The item difficulty parameter reflected how easy or difficult an item was to be endorsed by individuals with an average level of the trait. Overall craving, aggregated across all food items in the FCI, was calculated to account for in analyses, and may be considered to represent trait-level craving. The PCM was conducted to determine which food items in the FCI were more easily or commonly craved than other food items when individuals had the same level of overall food craving. The IRT parameters were estimated using marginal maximum likelihood (MML) with the expected and maximization (EM) algorithm (Lord, 1986).

Before estimating item parameters, the unidimensionality assumption of the construct was examined using principal component analysis (PCA). Next, item parameters (i.e., item difficulty) were estimated. Finally, DIF analysis was conducted to assess whether there were systematic differences in item responses between male and female participants while accounting for their underlying levels of food craving. The results of the DIF analysis provide insights into whether certain food items from the FCI function differently males and females, which can more accurately reflect sex differences in craving for specific food items. The results also inform whether higher latent (overall) craving may be related to differences in craving for different food items across males and females.

DIF analysis was conducted based on the multigroup IRT approach (Bock and Zimowski, 1997) and DIF detection procedure was conducted with a sequential-free baseline procedure (Chun et al., 2016) (detailed in the Supplementary material). Lastly, the effect sizes of true DIF items were calculated following Meade's (2010) approach, which provides a standardized item characteristic difference between two groups for DIF items and is comparable to conventional effect size measures (i.e., Cohen's D). Applied to our study, the effect size for DIF items indicated which food items showed greater ease to crave across males and females, given the same level of overall food craving. More details on the DIF detection procedure are provided in the Supplementary material.

## 3 Results

### 3.1 Descriptive statistics

Demographic information for the sample is presented in Table 1. The average age of the sample was 37.9 years, and the average BMI was 26.5, calculated from participants' self-report of height and weight. The sample was comprised of 52.7% males. Descriptive statistics for each item in the FCI are displayed in Supplementary Table 1.

**Table 1:** Demographic characteristics (*N* = 583).

**Variable**	**Male (*n* = 308) Mean/*N* (SD/%)**	**Female (*n* = 275) Mean/*N* (SD/%)**
Age	36.28 (9.95)	39.28 (11.37)
**Race**		
White	238 (77.27%)	216 (78.55%)
Black or African American	26 (8.44%)	20 (7.27%)
Asian	32 (10.39%)	18 (6.55%)
Native American or Alaskan Native	4 (1.30%)	5 (1.82%)
Native Hawaiian or other Pacific Islander	2 (0.65%)	6 (2.18%)
Multi-Racial	6 (1.95%)	10 (3.64%)
**Ethnicity - Hispanic/Latino**		
Yes	22 (7.14%)	24 (8.73%)
No	286 (92.86%)	251 (91.27%)
**BMI (calculated**	26.76 (7.10)	26.58 (7.36)
**from self-reported**		
**height and weight)**		

### 3.2 Unidimensionality assumption check for the FCI

According to the results from PCA, the first principal component explained 34.62% of the total variance, and the second principal component explained 8.77%. The first component accounted for around 4 times more of the total variance than the second component, which supports the unidimensionality of the FCI (Reckase, 1997).

### 3.3 Item characteristics of the FCI

The PCM model results facilitated a ranking of FCI items from the easiest to most difficult to crave as assessed across all participants in the sample (see Supplementary Table 2). Across participants, the easiest items to crave were pizza, French fries, and chocolate (item difficulty parameters ranged from 1.85 to 0.77), whereas the least craved food items were gravy, corn bread, and sausage (item difficulty parameters ranged from 2.36 to 3.01). Item score plots, test information plots, and measurement error plot are presented in Supplementary Figures 1, 2. The information and measurement error plots indicated that the FCI more accurately measured overall food craving for individuals with relatively higher food craving levels compared to those with lower levels.

### 3.4 Differential item functioning by sex

**Figure 1:**
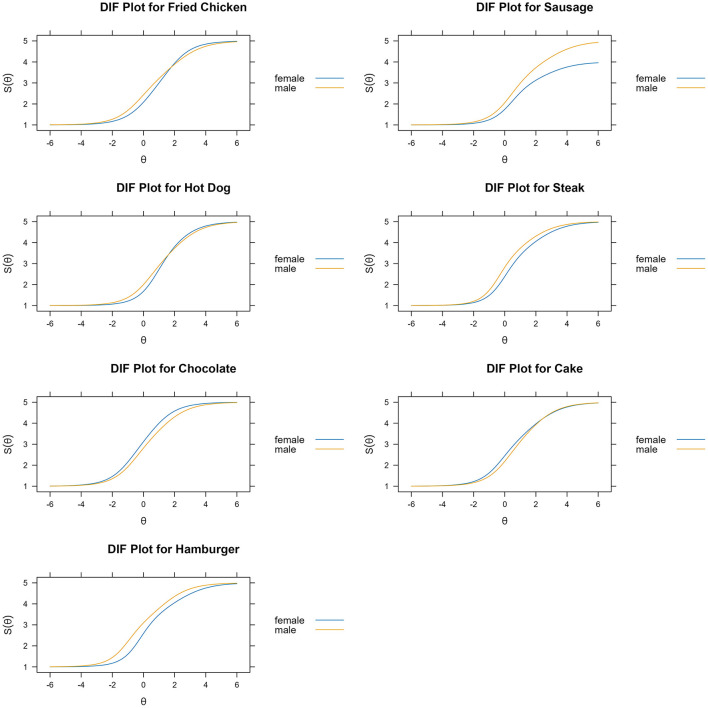
Item characteristic curves for items identified as differential functioning by sex. y-axis represents predicted score of craving for the specific food item. x-axis represents overall food craving. Higher values on the y-axis represents a greater expected score of craving for a specific food item at the respective θ levels on the x-axis (overall food craving). Yellow curve presents male's expected score along the overall food craving levels. Blue curve denotes female's expected score along the overall food craving levels.

According to the DIF analysis, the following items were identified as DIF: item 1 (fried chicken), item 2 (sausage), item 7 (hot dog), item 8 (steak), item 12 (chocolate), item 14 (cake), and item 25 (hamburger). The findings suggested that fried chicken, sausage, hot dogs, and hamburgers were harder for females to crave, whereas chocolate and cake were harder for males to crave, despite the groups having the same overall level of food craving (Table 2). DIF items identified as being easier for males to crave (savory items) had medium effect sizes ranging from 0.53 (hot dog) to 0.80 (hamburger). DIF items that were easier for females to crave (sweet items) had effect sizes ranging from 0.42 (cake) to 0.49 (chocolate). Regarding latent (overall) food craving, DIF analysis indicated that females needed a higher level of latent food craving to report cravings for savory items identified as DIF compared to males. In contrast, males required a higher level of food craving to endorse cravings for chocolate and cake compared to females. Thus, findings suggested that females who craved savory items had higher average craving, and males who craved sweet items had higher average craving. Item scale plots for each DIF item depending on sex are presented in Figure 1. For more detailed results of DIF detection, please see Supplementary Table 3.

**Table 2:** Results from the free base line models for DIF in sex.

**Items**	**SABIC**	**Chi-square**	***P*-value**	**Adjusted *P*-value**	**Effect size (Cohen's D)**
FCI 1 (Fried chicken)	−6.097	18.885	0.001	0.001	0.577
FCI 2 (Sausage)	−15.469	28.257	< 0.000	< 0.000	0.632
FCI 7 (Hotdog)	−4.208	16.996	0.002	0.002	0.517
FCI 8 (Steak)	−6.258	19.046	0.001	0.001	0.559
**FCI 12 (Chocolate)**	–**4.181**	**16.969**	**0.002**	**0.002**	**0.480**
**FCI 14 (Cake)**	**3.197**	**9.590**	**0.048**	**0.048**	**0.400**
FCI 25 (Hamburger)	−27.995	40.783	< 0.000	< 0.000	0.811

Bold items are DIF items that were easier for females to crave, given the same food craving level overall. Unbolded items represent DIF items that were easier for males to crave.

## 4 Discussion

Traditionally in the literature, food craving has been studied as a general construct and characterized by aggregating craving ratings across different types of foods to obtain a total score. However, specific foods may differently influence craving intensity across individuals and among different groups (e.g., males and females), which is important to understand from a risk and prevention perspective. The current study evaluated variability in craving across different types of foods among a general sample of adults. Additionally, the study overcame the limitations of previous studies on sex differences in craving by examining differences in craving for specific foods across males and females, while holding average level of craving constant. The results revealed substantial variability in the degree of craving intensity for different foods, suggesting that individuals may differ in craving for different food types. Furthermore, findings revealed differences in the types of foods craved across males and females, even when holding average craving level constant; savory food items were more easily (commonly) craved by males, whereas sweet items were more easily craved by females. Taken together, this study highlighted that food craving may vary depending on the type of food and sex.

One key finding of the study was that food craving varied substantially when examined at the food item-level. Specifically, pizza, French fries, and chocolate were the easiest (most common) to crave, whereas gravy, cornbread, and sausage were the hardest. The items that were most commonly craved are foods that are commonly consumed among the US population, whereas the foods that were the hardest to crave are less commonly consumed nationally (U. S. Department of Agriculture, Agricultural Research Service, 2023). Thus, the findings appear to reflect common dietary intake of the US population. The findings may also reflect the wide availability of some HPF in the US food environment, which could contribute to cue-induced cravings and subsequent intake. Pizza and French fries are widely available and commonly consumed in the US food environment across various settings, from fast food restaurants to frozen grocery options (Zenk et al., 2015). Similarly, chocolate is widely available and heavily marketed in a variety of contexts in the US (Sebastian et al., 2010). Thus, the high availability and marketing of HPF in the US food supply may contribute to cue-induced cravings through repeated exposure and classical conditioning that associate these foods with rewarding experiences (Hill, 2007; Rejeski et al., 2010; Boyland et al., 2024). In contrast, food items like gravy may be less commonly encountered at the national scale, and may be more event- or culture-specific (e.g., gravy served with meat at holidays), and thus may not trigger cravings as readily. Overall, our results suggested that the most commonly craved food items were HPF that are popular and commonly consumed in the US, and thus may reflect their extensive availability in the US food environment.

The DIF analysis revealed moderate to large effect size differences in cravings for specific foods by sex, with males finding certain savory foods easier to crave and females finding some sweet foods easier to crave. Importantly, these findings emerged despite overall food craving across males and females being held constant in analyses; therefore, the findings were able to disentangle the effects of specific food items from overall craving level across males and females. Previous studies have attempted to test sex differences in craving intensity using single food items, with chocolate being a common food stimulus. Studies typically observed higher craving intensity ratings for chocolate among females relative to males (Hormes et al., 2014; Meule and Hormes, 2015), which appear to have influenced the focus on female samples in research on chocolate craving (Tapper and Turner, 2018; Meule et al., 2019; Richard et al., 2019) and overall craving research (Richard et al., 2017; Zorjan and Schienle, 2023). However, our findings suggest that these studies may have selected a food stimulus that females tend to crave more commonly or easily despite having relatively lower overall food craving than males, which may have impacted the findings and the interpretations of sex differences. In contrast, if a study had selected a savory item such as hamburger, the findings may have indicated higher craving intensity among males and may have resulted in an interpretation that males experience more craving relative to females. Thus, our finding highlights the importance of considering the item-specific nature of cravings when examining differences in craving intensity across groups.

The results of the DIF analysis may provide nuanced insights into craving across sexes, both regarding types of foods craved and also level of overall craving. Our study was conducted in a general sample of adults and therefore we cannot make inferences regarding clinical risk. However, our study identified distinct patterns of craving across sexes that also coincided with higher overall craving. Future research replicating this work among clinical samples, including individuals with elevated craving, obesity, and/or disordered eating could inform whether the sex-specific food item cravings identified in the study, combined with higher overall craving, may be a risk factor for clinical conditions.

The study had several limitations. First, the sample may not be representative of the broader US population, particularly in terms of race and ethnicity. Future studies should aim to include more racially and ethnically diverse populations to improve the generalizability of the findings. Second, the study did not assess where participants lived in the US, which could have influenced the results, especially given that cornbread was identified as particularly difficult to crave. Future studies should examine the potential effects of region on cravings for different types of foods. Third, the study measured sex (male, female) but also included an option for transgender, which conflated gender identity in the survey item. Although most participants (99.5%; 583/585) endorsed sex as male or female, *n* = 2 individuals selected the option for transgender. Because the sample that endorsed transgender was too small to analyze as a group, we did not include the participants in analyses. However, this approach may limit the generalizability of our findings to more diverse populations, including individuals who identify as transgender. Future studies should clearly distinguish questions for biological sex and gender identity to improve measurement of these constructs and to examine potential differences in food item level craving. Lastly, our study did not include eating-related variables such as dietary intake, which limits the contextualization of our findings within broader patterns of eating behavior. Future studies should examine how sex differences in cravings for specific food items relate to eating behaviors and dietary intake.

## 5 Conclusions and implications

The study findings highlight the nuances of food cravings, which differed by food items and sex, underscoring the need for more refined approaches in craving assessment research. The study identified distinct patterns of craving across sexes that also occurred with higher overall craving, and future work is needed to understand whether such presentations could be considered as risk factors for disordered eating and related clinical conditions.

## Data Availability

The raw data supporting the conclusions of this article will be made available by the authors upon reasonable request and approval of the institutional review board, without undue reservation.
