# Supplementary material for: Beyond general food craving: sex differences in food-specific craving identified using item response theory
Source: Front Psychol. 2025 Jun 17;16:1588999. doi: 10.3389/fpsyg.2025.1588999 (PMC12209352; doi:10.3389/fpsyg.2025.1588999)
Supplement: Supplementary file 1 [file Table_1.docx]

Supplementary Material

# Supplementary Information

**Differential Item Functioning Analysis**

DIF analysis was conducted based on the multigroup IRT approach (Bock & Zimowski, 1997) and DIF detection procedure was conducted with a sequential-free baseline procedure (Chun et al., 2016). The sequential-free baseline procedure consists of two steps to identify DIF. Specifically, in the first step, two competing models were estimated: constrained versus free models. For the constrained model, two groups’ item parameters were simultaneously estimated with the equality constraint from the multigroup IRT model. In other words, item parameters from both male and female groups were identically estimated. For the free model, two group’s item parameters were simultaneously estimated with the equality constraint except for a specific item (i.e., DIF-tested item). The DIF-tested item parameters were freely estimated in the model (i.e., free model). Finally, two models (constrained vs. free models) were tested based on the Likelihood Ratio Test (LRT). If two models showed significant difference, the DIF-tested item was considered as DIF; otherwise, the DIF-tested item was considered non-DIF. This DIF detection procedure (LRT for two models) continued for all items simultaneously for the FCI. In general, the sequential-free baseline DIF procedure moves on to the second step if at least one item from was identified as DIF from the measure; otherwise, the sequential-free baseline DIF procedure stops at the first step.

In the second step, again, two competing models were tested: free versus constrained model. For the free model, all item parameters were freely estimated across two groups except for the anchoring items (note that the anchoring items were non-DIF from the first step). For the constrained model, all items were freely estimated across two groups except for the anchoring items *and* a DIF-tested item. Similar to the first-step, two competing models were tested using LRT. If two models showed significant difference, the DIF-tested item was considered DIF; otherwise, the DIF-tested item was considered non-DIF. This procedure continued for remaining DIF-detected items from the first step. The sequential-free baseline DIF detection method has demonstrated better performance in controlling Type I errors and higher power to detect true DIF items compared to the other conventional DIF methods such as logistic regression (e.g., (Chun et al., 2016; Joo et al., 2022; Kim et al., 2016; Stark et al., 2006). To control the family-wise error rate, the Hochberg’s step-up procedure was implemented in the DIF analysis (Hochberg, 1988).

Finally, the effect sizes of true DIF items were calculated following Meade’s (2010) approach. This approach calculates effect size for DIF items by comparing the expected item scores between groups across the latent trait continuum. Specifically, the standardized difference of the expected item score’s probability distribution function were computed across the latent trait continuum. The DIF effect size provides a standardized item characteristic difference between two groups for DIF items and is comparable to conventional effect size measures (i.e., Cohen’s D). This approach facilitated comparisons of effect size across different items as well as an understanding of the practical significance of measurement invariance beyond statistical significance. Applied to our study, the effect size for DIF items indicated which food items showed greater ease to crave across males and females, given the same level of overall food craving.

# Supplementary Tables

**Supplemental** **Table S1. Descriptive Statistics of the Food Craving Inventory Stratified by Sex**

| Variable | Male (N = 308)  Mean (SD) | Female (N = 275)  Mean (SD) |
| --- | --- | --- |
| FCI 1 – friend chicken | 2.45 (1.03) | 2.11 (1.02) |
| FCI 2 – sausage | 2.15 (1.06) | 1.78 (0.90) |
| FCI 3 – gravy | 1.82 (0.98) | 1.61 (0.84) |
| FCI 4 – fried fish | 2.03 (1.07) | 1.89 (1.02) |
| FCI 5 – bacon | 2.63 (1.13) | 2.39 (1.12) |
| FCI 6 – corn bread | 1.83 (0.97) | 1.84 (1.01) |
| FCI 7 – hot dog | 2.09 (1.04) | 1.77 (1.00) |
| FCI 8 – steak | 2.79 (1.23) | 2.36 (1.36) |
| FCI 9 – brownies | 2.38 (1.08) | 2.44 (1.11) |
| FCI 10 – cookies | 2.73 (1.16) | 2.75 (1.07) |
| FCI 11 – candy | 2.45 (1.1) | 2.52 (1.10) |
| FCI 12 – chocolate | 2.81 (1.13) | 3.05 (1.15) |
| FCI 13 – donuts | 2.42 (1.07) | 2.41 (1.11) |
| FCI 14 – cake | 2.26 (1.09) | 2.41 (1.06) |
| FCI 5 – cinnamon rolls | 2.21 (1.09) | 2.12 (1.07) |
| FCI 16 – ice cream | 2.75 (1.13) | 2.79 (1.13) |
| FCI 17 – rolls | 2.24 (1.06) | 2.29 (1.1) |
| FCI 18 – pancake or waffles | 2.41 (1.04) | 2.35 (1.04) |
| FCI 19 – biscuits | 2.18 (1.05) | 2.08 (1.06) |
| FCI 20 – sandwich bread | 2.23 (1.16) | 2.16 (1.11) |
| FCI 21 – rice | 2.35 (1.18) | 2.23 (1.16) |
| FCI 22 – baked potato | 2.29 (1.15) | 2.23 (1.13) |
| FCI 23 – pasta | 2.75 (1.13) | 2.7 (1.12) |
| FCI 24 – cereal | 2.38 (1.2) | 2.24 (1.13) |
| FCI 25 – hamburger | 3.05 (1.08) | 2.52 (1.15) |
| FCI 26 – French fries | 3.1 (1.11) | 2.99 (1.09) |
| FCI 27 – chips | 2.68 (1.18) | 2.76 (1.17) |
| FCI 28 - pizza | 3.42 (1) | 3.14 (1.08) |

**Supplemental Table S2. Item Parameters from the PCM Model**

| FCI Items | b  (item difficulty) | d1 | d2 | d3 | d4 |
| --- | --- | --- | --- | --- | --- |
| FCI 1 – friend chicken | 1.09 | 0.38 | 0.3 | -1.26 | -3.78 |
| FCI 2 – sausage | 2.37 | -0.39 | -0.6 | -2.46 | -6.02 |
| FCI 3 – gravy | 3.2 | -0.78 | -1.42 | -3.52 | -7.08 |
| FCI 4 – fried fish | 2.16 | -0.39 | -0.83 | -2.37 | -5.05 |
| FCI 5 – bacon | 0.61 | 0.16 | 0.69 | -0.48 | -2.8 |
| FCI 6 – corn bread | 2.62 | -0.64 | -1.09 | -3.06 | -5.68 |
| FCI 7 – hot dog | 2.26 | -0.43 | -0.83 | -2.52 | -5.24 |
| FCI 8 – steak | 0.57 | -0.13 | 0.52 | -0.2 | -2.48 |
| FCI 9 – brownies | 0.78 | 0.35 | 0.51 | -0.82 | -3.15 |
| FCI 10 – cookies | -0.2 | 0.69 | 1.31 | 0.36 | -1.58 |
| FCI 11 – candy | 0.53 | 0.5 | 0.68 | -0.52 | -2.77 |
| FCI 12 – chocolate | -0.75 | 0.95 | 1.67 | 0.97 | -0.58 |
| FCI 13 – donuts | 0.73 | 0.37 | 0.58 | -0.88 | -3 |
| FCI 14 – cake | 1.05 | 0.21 | 0.32 | -1.07 | -3.66 |
| FCI 5 – cinnamon rolls | 1.61 | -0.12 | -0.19 | -1.6 | -4.53 |
| FCI 16 – ice cream | -0.32 | 0.81 | 1.32 | 0.5 | -1.33 |
| FCI 17 – rolls | 1.35 | 0.02 | 0.12 | -1.28 | -4.28 |
| FCI 18 – pancake or waffles | 0.84 | 0.48 | 0.58 | -0.9 | -3.54 |
| FCI 19 – biscuits | 1.75 | -0.19 | -0.2 | -1.85 | -4.76 |
| FCI 20 – sandwich bread | 1.49 | -0.28 | -0.21 | -1.59 | -3.88 |
| FCI 21 – rice | 1.21 | -0.18 | -0.07 | -1.19 | -3.41 |
| FCI 22 – baked potato | 1.39 | -0.25 | -0.06 | -1.29 | -3.96 |
| FCI 23 – pasta | 0 | 0.5 | 1.15 | 0.43 | -2.09 |
| FCI 24 – cereal | 1.03 | 0.12 | -0.06 | -1.19 | -2.99 |
| FCI 25 – hamburger | -0.09 | 0.24 | 1.28 | 0.5 | -1.67 |
| FCI 26 – French fries | -0.9 | 0.69 | 1.93 | 1.48 | -0.5 |
| FCI 27 – chips | -0.12 | 0.66 | 1.03 | 0.23 | -1.45 |
| FCI 28 - pizza | -1.83 | 1.26 | 2.7 | 2.47 | 0.91 |

Note. d1:d4 indicates threshold parameters. Item difficulty parameter (b) was reparametrized using threshold parameter following this formula: b = -(d1 + d2 + d3 + d4)/4.

**Supplemental Table S3. Results of the Constrained Model for DIF in Sex**

| FCI Item | SABIC | X2 | p-value | adjusted  p-value |
| --- | --- | --- | --- | --- |
| **FCI 1 – friend chicken** | -3.063 | 15.851 | 0.003 | **0.019*** |
| **FCI 2 – sausage** | -12.572 | 25.36 | < .001 | **0.001*** |
| FCI 3 – gravy | 5.48 | 7.308 | 0.12 | 0.211 |
| FCI 4 – fried fish | 9.153 | 3.635 | 0.458 | 0.583 |
| FCI 5 – bacon | 9.048 | 3.74 | 0.442 | 0.583 |
| FCI 6 – corn bread | 4.723 | 8.065 | 0.089 | 0.211 |
| **FCI 7 – hot dog** | -1.482 | 14.269 | 0.006 | **0.028*** |
| **FCI 8 – steak** | -2.926 | 15.713 | 0.003 | **0.019*** |
| FCI 9 – brownies | 4.832 | 7.955 | 0.093 | 0.211 |
| FCI 10 – cookies | 5.165 | 7.623 | 0.106 | 0.211 |
| FCI 11 – candy | 5.359 | 7.429 | 0.115 | 0.211 |
| **FCI 12 – chocolate** | -10.438 | 23.226 | < .001 | **0.001*** |
| FCI 13 – donuts | 7.312 | 5.476 | 0.242 | 0.388 |
| **FCI 14 – cake** | -1.311 | 14.099 | 0.007 | **0.028*** |
| FCI 5 – cinnamon rolls | 12.482 | 0.306 | 0.989 | 0.989 |
| FCI 16 – ice cream | 5.415 | 7.373 | 0.117 | 0.211 |
| FCI 17 – rolls | 5.154 | 7.633 | 0.106 | 0.211 |
| FCI 18 – pancake or waffles | 11.332 | 1.456 | 0.834 | 0.899 |
| FCI 19 – biscuits | 9.644 | 3.144 | 0.534 | 0.638 |
| FCI 20 – sandwich bread | 7.396 | 5.392 | 0.249 | 0.388 |
| FCI 21 – rice | 10.103 | 2.685 | 0.612 | 0.685 |
| FCI 22 – baked potato | 9.722 | 3.066 | 0.547 | 0.638 |
| FCI 23 – pasta | 8.274 | 4.514 | 0.341 | 0.477 |
| FCI 24 – cereal | 7.811 | 4.977 | 0.29 | 0.427 |
| **FCI 25 – hamburger** | -24.332 | 37.12 | < .001 | **< .001*** |
| FCI 26 – French fries | 12.034 | 0.753 | 0.945 | 0.98 |
| FCI 27 – chips | 4.398 | 8.39 | 0.078 | 0.211 |
| FCI 28 - pizza | 1.764 | 11.024 | 0.026 | 0.092 |

The LRT-based DIF analysis was conducted to examine potential item bias in the FCI between males and females. Based on the constrained model, 21 items were identified as non-DIF items (see Supplementary Table S3) from the first step of the sequential-free baseline DIF procedure. In the second step (i.e., free model), the 21 items were served as anchoring items in the model, and tested for DIF for remaining items. *Note*. Bolded items are potential DIF items (adjusted p-value < .05). The rest of the items are anchoring items. *** < .05**

# Supplementary Figures

**
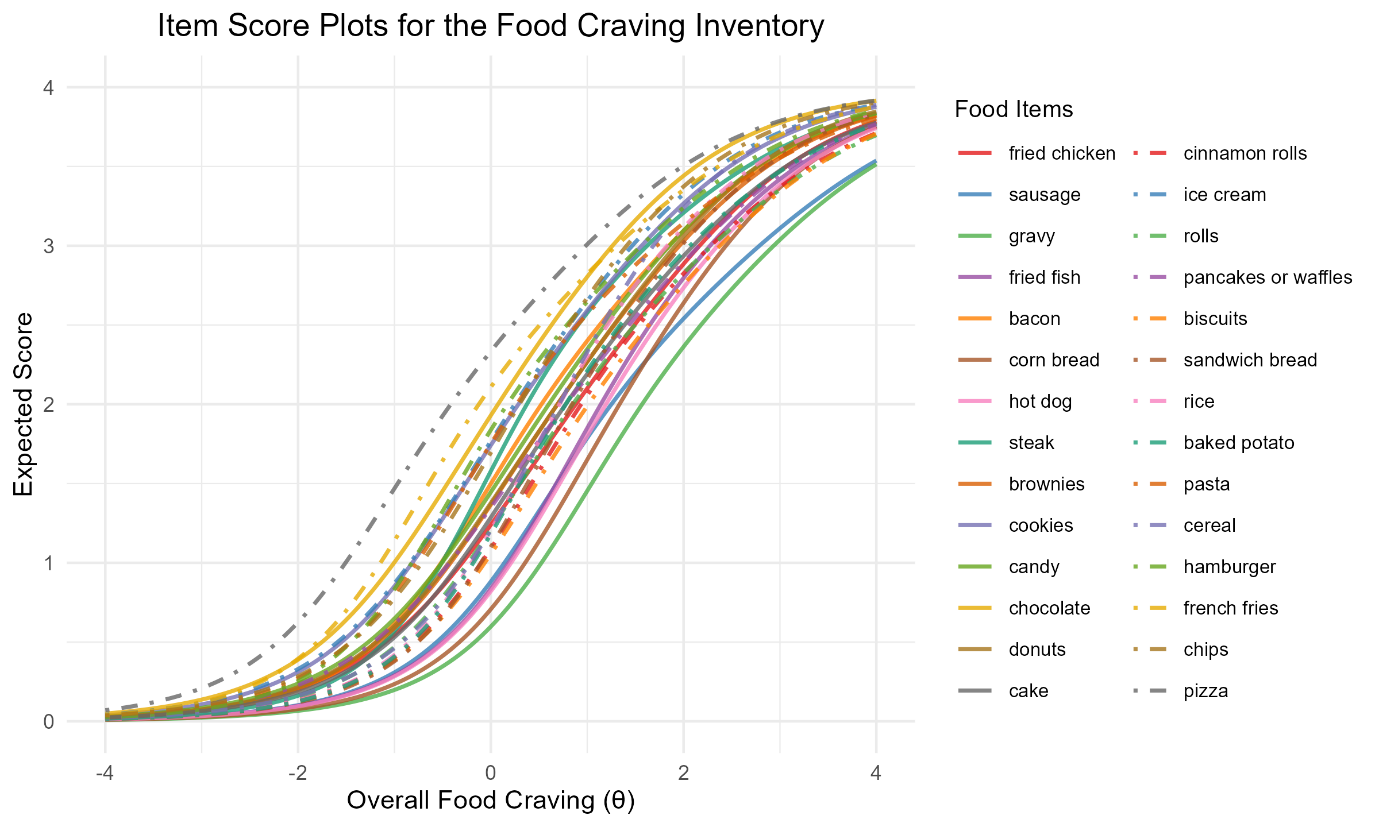
**

**Supplemental Figure S1. Item Score Plots for the PCM model of the Food Craving Inventory.** Supplemental Figure S1 presents item score plots for the food items in the Food Craving Inventory. Y-axis represents the expected score of craving for specific food items, and X-axis represents overall food craving level. Higher values on the y-axis indicates greater expected score of craving for a specific food at the respective θ levels on the x-axis (overall food craving).

**
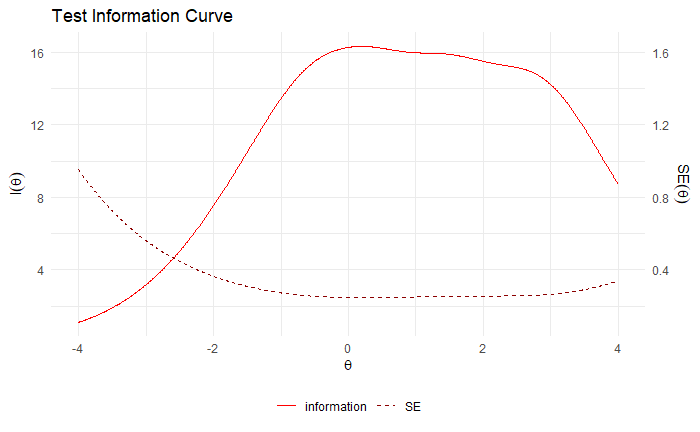
**

**Supplemental Figure S2. Test Information Curve Plot for the PCM model of the Food Craving Inventory.** Supplemental Figure S2 presents test information curve and measurement error plot for the Food Craving Inventory. X-axis represents overall food craving levels produced by the PCM model. Y-axis on the left-side represents the test information, and Y-axis on the right-side represents the measurement error at the respective θ levels on the x-axis. Higher values on the y-axis indicate greater test information and lower measurement error at the respective the X-axis value.
